# TKSM: highly modular, user-customizable, and scalable transcriptomic sequencing long-read simulator

**DOI:** 10.1093/bioinformatics/btae051

**Published:** 2024-01-25

**Authors:** Fatih Karaoğlanoğlu, Baraa Orabi, Ryan Flannigan, Cedric Chauve, Faraz Hach

**Affiliations:** Computing Science Department, Simon Fraser University, Burnaby, BC V5A 1S6, Canada; Department of Computer Science, the University of British Columbia, Vancouver, BC V6T 1Z4, Canada; Department of Urologic Sciences, the University of British Columbia, Vancouver, BC V5Z 1M9, Canada; Vancouver Prostate Centre, Vancouver, BC V6H 3Z6, Canada; Department of Mathematics, Simon Fraser University, Burnaby, BC V5A 1S6, Canada; Department of Computer Science, the University of British Columbia, Vancouver, BC V6T 1Z4, Canada; Department of Urologic Sciences, the University of British Columbia, Vancouver, BC V5Z 1M9, Canada; Vancouver Prostate Centre, Vancouver, BC V6H 3Z6, Canada

## Abstract

**Motivation:**

Transcriptomic long-read (LR) sequencing is an increasingly cost-effective technology for probing various RNA features. Numerous tools have been developed to tackle various transcriptomic sequencing tasks (e.g. isoform and gene fusion detection). However, the lack of abundant gold-standard datasets hinders the benchmarking of such tools. Therefore, the simulation of LR sequencing is an important and practical alternative. While the existing LR simulators aim to imitate the sequencing machine noise and to target specific library protocols, they lack some important library preparation steps (e.g. PCR) and are difficult to modify to new and changing library preparation techniques (e.g. single-cell LRs).

**Results:**

We present TKSM, a modular and scalable LR simulator, designed so that each RNA modification step is targeted explicitly by a specific module. This allows the user to assemble a simulation pipeline as a combination of TKSM modules to emulate a specific sequencing design. Additionally, the input/output of all the core modules of TKSM follows the same simple format (Molecule Description Format) allowing the user to easily extend TKSM with new modules targeting new library preparation steps.

**Availability and implementation:**

TKSM is available as an open source software at https://github.com/vpc-ccg/tksm.

## 1 Introduction

Long-read (LR) sequencing technologies have become a cost-effective alternative to short-read (SR) sequencing for many genomic and transcriptomic sequencing tasks (Amarasinghe *et al.* 2020). LRs are shown to be useful for many transcriptomic tasks such as alternative isoform detection (Kovaka *et al.* 2019, Tang *et al.* 2020, Orabi *et al.* 2023), gene fusion detection (Liu *et al.* 2020, Karaoglanoglu *et al.* 2022), transcript-level expression analysis (Hu *et al.* 2021), or single-cell transcriptomic analysis (Tian *et al.* 2021, Ebrahimi *et al.* 2022, You *et al.* 2023).

However, due to the nature of LR sequencing as an emerging technology, there are very few well established benchmark datasets or gold-standard datasets to assess transcriptomic LR bioinformatics tools. Such bioinformatics tools targeting these tasks require realistic simulations in order to assess their accuracy and performance. This includes the ability to simulate explicitly target specific library or cellular processes such as single-cell barcoding and UMI tagging, PCR, or molecule truncation.

Existing LR simulators such as Badread (Wick 2019), DeepSimulator (Li *et al.* 2020), Icarust (Munro *et al.* 2023), PBSIM3 (Ono *et al.* 2022), and Nanosim (Yang *et al.* 2017), typically focus on simulating the sequencing process, i.e., the point of contact of sequencing platform with the RNA/DNA molecule. Some have extensions focusing on specific sequencing libraries such as Trans-Nanosim transcriptomic and plasmid simulation (Hafezqorani *et al.* 2020), Meta-Nanosim metagenomic simulation (Yang *et al.* 2023), SLSim single-cell simulation (You *et al.* 2023a), and SQANTI-SIM alternative splicing simulation (Mestre-Tomás *et al.* 2023). However, these tools are not designed with modularity in mind and cannot be easily modified to address changes in the library preparation protocols such as adding a barcode tag or simulating the PCR process. A comprehensive survey of long-read tools, including simulation tools, is available at Long-read Tools catalogue (Amarasinghe *et al.* 2021).

We describe TKSM, a software that simulates realistic transcriptomic long-read datasets. TKSM modular design allows to target a wide range of library/cell processes. The power of TKSM lies in two key aspects: (i) the ease with which its simulation pipeline can be modified to cater to specific sequencing designs and (ii) high performance in terms of time and memory use. TKSM is open source, accessible via GitHub.

## 2 Methods

TKSM is flexible, both in that it can simulate a wide variety of datasets, and it is extendable. It is composed of several independent modules, each representing a cellular (e.g. polyadenylation) or a library preparation (e.g. PCR) process that modifies a nucleic acid molecule. This design allows the user to simulate different sequencing protocols by using TKSM’s modules in various arrangements, imitating the different steps in the desired sequencing protocol. Additionally, this modular design allows TKSM to be easily extendable with future modules targeting additional library and cellular processes. To enable this modularity, we designed TKSM’s modules to take and generate files in the same format that we call Molecule Description Format (MDF). An MDF file is a tabular file that describes molecules by listing for each molecule its genomic intervals alongside any sequence-level modifications to these intervals (e.g. substitutions). The rationale for using a tabular format is that write their own scripts that can generate or modify intermediate MDF files. We expand on the details of MDF files in Supplementary Section S1. The only exceptions to this design pattern are the entry module which generates the initial set of molecules from a transcript abundance profile and the exit module which generates the reads obtained by simulating the sequencing of the given molecules.

Each of TKSM’s modules can be run as a separate process (tksm < module_name >). We also provide as part of TKSM a Snakemake (Mölder *et al.* 2021) script which can be configured by the user to specify a wide range of simulation experiments and run them all as a single command. Additionally, to optimize the computation time, we take advantage of Snakemake’s piped input/output feature to allow modules to start running the moment they receive any input from a previous module, rather than having to wait for the preceding module to terminate.

TKSM can use real sequencing datasets to parameterize the behaviour of its modules, or alternatively, these parameters can be specified manually by the user. For example, TKSM contains preprocessing modules to compute the expression profile of transcripts from a given real sample which is then used to generate the molecules in the initial MDF file, whose sequencing according to a chosen protocol will be simulated by the next modules.

### 2.1 TKSM modules

**Figure 1.**
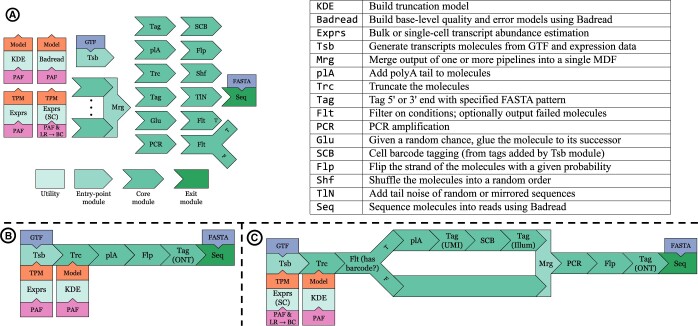
(A) Existing TKSM modules and utilities alongside their high-level descriptions. TKSM is designed with modularity in mind; the user can specify a simulation pipeline of their choosing by chaining any number of TKSM modules including the possibility of using the same module multiple times. (B) Typical RNA-seq simulation pipeline that imitates Trans-Nanosim’s workflow. (C) Single-cell long-read simulation pipeline. The pipeline makes use of the Filtering and Merging modules to add the short-read Illumina adapter and 10× Genomics cellular barcodes only to molecules that have a tag indicating that they should have a cellular barcode.

TKSM contains three classes of modules, defined by features of their input and output: (i) entry-point modules start a TKSM pipeline and output an MDF file, (ii) core modules take an MDF file as input and output another MDF file, and (iii) exit (sequencing) modules take an MDF file as input and generate FASTA/FASTQ file(s) as output. Additionally, some preprocessing utilities in TKSM can take a real sequencing dataset and output model parameters for some of TKSM modules. A list of the implemented TKSM modules is presented in Fig. 1A and detailed in Supplementary Section S1. Additional modules and utilities can be implemented and easily integrated into TKSM in order to target specific steps in alternate sequencing protocols.

### 2.2 Customizable TKSM pipelines using Snakemake

An important design choice for TKSM is to make it easily customizable by the user, i.e. to make it easy to build a, possibly complex, simulation pipeline using the TKSM modules. To achieve that, we packaged TKSM with Snakemake and configuration scripts that can be edited by the user to add new modules or to define simulation experiments using any arrangement of TKSM modules. To define a simulation pipeline, the user lists the names of required TKSM modules and specifies, for the modules that require model construction, the real samples to build such models on. Additionally, using the Merging module, the user may build complex pipelines that are composed of different linear pipelines. An example of the configuration script is presented in Supplementary Listing S1.

## 3 Results

To illustrate TKSM and assess its performances, we designed three simulation pipelines to emulate examples of standard transcriptomic sequencing protocols. Specifically, we present simulations of a standard bulk RNA sequencing experiment, a hybrid long-short read single-cell RNA sequencing (scRNA-seq) experiment, and an RNA sequencing experiment similar to the bulk RNA sequencing experiment but with 100 random gene fusion events added. The Snakemake configuration files that specify these simulation pipelines are presented in Supplementary Listings S2–S4.

In the standard bulk RNA-seq experiment, we primarily compare against Trans-Nanosim (Hafezqorani *et al.* 2020) and try to conform to its pipeline design using TKSM modules. For both the bulk and gene fusion experiments, we use an RNA-seq sample generated from the MCF7 cell line by Chen *et al.* (2021) (direct RNA, replicate 1, run 2). We first accessed the SG-NEx data on 2020–06-17 via https://registry.opendata.aws/sgnex/. For the scRNA-seq experiment, we used an in-house dataset, named N1, first described by Ebrahimi *et al.* (2022). N1 follows the short-long single-cell hybrid protocol described previously in the literature (Gupta *et al.* 2018, Singh *et al.* 2019, Tian *et al.* 2021). In this manuscript, we use a random subsample of N1 with ∼1M long-reads. The three TKSM pipelines are illustrated in Fig. 1B and C and Supplementary Figure S11.

Using these experiments, our goal is to assess TKSM on multiple metrics: (i) the similarity of the simulated data compared to the input real data on measures such as transcript expression, molecule sequence truncation, single cell barcode detection rates, and gene fusion generation, (ii) the time and memory footprint of various steps, and (iii) the ability to generate gene fusion events that can be detected by standard gene fusion tools. The results of all these experiments are presented in Supplementary Section S2. Note that all these results are reproducible using Snakemake scripts provided on the TKSM GitHub repository.

## 4 Conclusion

TKSM is a modular, accurate, and efficient transcriptomic LR sequencing simulator. Its modular design enables the user to construct a large verity of sequencing experiments with minimal effort. TKSM’s standardized input and output for its modules allow the users of TKSM to add new modules that target existing and future library preparation techniques that TKSM currently does not target. For example, it is easy to envision an alternative entry-point module to the Transcribing module that generates nucleic acid molecules from DNA fragmentation while still making use of the rest of TKSM modules. TKSM also performs well in terms of generating realistic datasets with characteristics matching the real datasets it is simulating. Additionally, TKSM is engineered with efficient CPU and memory use in mind and its performance on those metrics is excellent.

## Supplementary Material

btae051_Supplementary_Data

## Data Availability

TKSM is available as an open source software at github.com/vpc-ccg/tksm. Datasets used in this manuscript are publicly available via registry.opendata.aws/sgnex/for the MCF7 dataset and at doi.org/10.6084/m9.figshare.23155145 for the N1 sample. An archive of the TKSM version used in this manuscript is available at doi.org/10.6084/m9.figshare.24970317.v1.

## References

[btae051-B1] Amarasinghe SL , Su S, Dong X et al Opportunities and challenges in long-read sequencing data analysis. Genome Biol 2020;21:30–16. 10.1186/s13059-020-1935-5. 32033565 PMC7006217

[btae051-B2] Amarasinghe SL , Ritchie ME, Gouil Q. long-read-tools.org: an interactive catalogue of analysis methods for long-read sequencing data. GigaScience 2021;10. 10.1093/gigascience/giab003 PMC7931822 33590862

[btae051-B3] Chen Y , Davidson NM, Wan YK et al A systematic benchmark of nanopore long read RNA sequencing for transcript level analysis in human cell lines. bioRxiv, 10.1101/2021.04.21.440736, 2021, preprint: not peer reviewed. PMC11978509 40082608

[btae051-B4] Ebrahimi G , Orabi B, Robinson M et al Fast and accurate matching of cellular barcodes across short-reads and long-reads of single-cell RNA-seq experiments. iScience 2022;25:104530. 10.1016/j.isci.2022.104530. 35747387 PMC9209721

[btae051-B5] Gupta I , Collier PG, Haase B et al Single-cell isoform RNA sequencing characterizes isoforms in thousands of cerebellar cells. Nat Biotechnol 2018;36:1197–202. 10.1038/nbt.4259. 30320766

[btae051-B6] Hafezqorani S , Yang C, Lo T et al Trans-NanoSim characterizes and simulates nanopore RNA-sequencing data. GigaScience 2020;9. 10.1093/gigascience/giaa061. PMC7285873 32520350

[btae051-B7] Hu Y , Fang L, Chen X et al LIQA: long-read isoform quantification and analysis. Genome Biol 2021;22:182. 10.1186/s13059-021-02399-8. 34140043 PMC8212471

[btae051-B8] Karaoglanoglu F , Chauve C, Hach F. Genion, an accurate tool to detect gene fusion from long transcriptomics reads. BMC Genomics 2022;23:129. 10.1186/s12864-022-08339-5. 35164688 PMC8842519

[btae051-B9] Kovaka S , Zimin AV, Pertea GM et al Transcriptome assembly from long-read RNA-seq alignments with StringTie2. Genome Biol 2019;20:278–13. 10.1186/s13059-019-1910-1. 31842956 PMC6912988

[btae051-B10] Li Y , Wang S, Bi C et al DeepSimulator1.5: a more powerful, quicker and lighter simulator for nanopore sequencing. Bioinformatics 2020;36:2578–80. 10.1093/bioinformatics/btz963. 31913436 PMC7178411

[btae051-B11] Liu Q , Hu Y, Stucky A et al LongGF: computational algorithm and software tool for fast and accurate detection of gene fusions by long-read transcriptome sequencing. BMC Genomics 2020;21:793–12. 10.1186/s12864-020-07207-4. 33372596 PMC7771079

[btae051-B12] Mestre-Tomás J , Liu T, Pardo-Palacios F et al SQANTI-SIM: a simulator of controlled transcript novelty for lrRNA-seq benchmark. bioRxiv 2023.08.23.554392, preprint: not peer reviewed. 10.1186/s13059-023-03127-0 PMC10712166 38082294

[btae051-B13] Mölder F , Jablonski KP, Letcher B et al Sustainable data analysis with Snakemake. F1000Res 2021;10:33. 10.12688/f1000research.29032.2. 34035898 PMC8114187

[btae051-B14] Munro RJ , Payne A, Loose MW. Icarust, a real-time simulator for Oxford Nanopore adaptive sampling. bioRxiv 2023.05.16.540986, preprint: not peer reviewed. 10.1093/bioinformatics/btae141 PMC10980563 38478392

[btae051-B15] Ono Y , Hamada M, Asai K. PBSIM3: a simulator for all types of PacBio and ONT long reads. NAR Genom Bioinform 2022;4:. 10.1093/nargab/lqac092. PMC9713900 36465498

[btae051-B16] Orabi B , Xie N, McConeghy B et al Freddie: annotation-independent detection and discovery of transcriptomic alternative splicing isoforms using long-read sequencing. Nucleic Acids Res 2023;51:e11. 10.1093/nar/gkac1112. 36478271 PMC9881145

[btae051-B17] Singh M , Al-Eryani G, Carswell S et al High-throughput targeted long-read single cell sequencing reveals the clonal and transcriptional landscape of lymphocytes. Nat Commun 2019;10:3120–13. 10.1038/s41467-019-11049-4. 31311926 PMC6635368

[btae051-B18] Tang AD , Soulette CM, van Baren MJ et al Full-length transcript characterization of SF3B1 mutation in chronic lymphocytic leukemia reveals downregulation of retained introns. Nat Commun 2020;11:1438. 10.1038/s41467-020-15171-6. 32188845 PMC7080807

[btae051-B19] Tian L , Jabbari JS, Thijssen R et al Comprehensive characterization of single-cell full-length isoforms in human and mouse with long-read sequencing. Genome Biol 2021;22:310–24. 10.1186/s13059-021-02525-6. 34763716 PMC8582192

[btae051-B20] Wick RR. Badread: simulation of error-prone long reads. JOSS 2019;4:1316. 10.21105/joss.01316.

[btae051-B21] Yang C , Chu J, Warren RL et al NanoSim: nanopore sequence read simulator based on statistical characterization. GigaScience 2017;6:1–6. 10.1093/gigascience/gix010 PMC5530317 28327957

[btae051-B22] Yang C , Lo T, Nip KM et al Characterization and simulation of metagenomic nanopore sequencing data with Meta-Nanosim. GigaScience 2023;12:giad013. 36939007 10.1093/gigascience/giad013 PMC10025935

[btae051-B23] You Y , Prawer YD, De Paoli-Iseppi R et al Identification of cell barcodes from long-read single-cell RNA-seq with BLAZE. Genome Biol 2023;24:66. 37024980 10.1186/s13059-023-02907-y PMC10077662

