# Supplementary material for: TKSM: highly modular, user-customizable, and scalable transcriptomic sequencing long-read simulator
Source: Bioinformatics. 2024 Jan 25;40(2):btae051. doi: 10.1093/bioinformatics/btae051 (PMC10868325; doi:10.1093/bioinformatics/btae051)
Supplement: btae051_Supplementary_Data [file btae051_supplementary_data.pdf]

Supplementary Material for TKSM

Fatih Karaoglanoglu Baraa Orabi Ryan Flannigan Cedric Chauve
Faraz Hach

S1 Supplementary Methods

In this section, we describe in detail TKSM's methodology including the new file format we use as a medium between TKSM's modules as well as the inner workings of each of TKSM's modules.

S1.1 Molecule Description Format

Each MDF entry (A molecule description) begins with a header line which consists of `+` symbol, followed by `<molecule_id>`, `<molecule_count>` and `<molecule_info>`:

```
+molecule_1 1 info1=1,2,3,4;info2;
```

This molecule header line is parsed by TKSM to:

```
{
 "Id": "molecule_1",
 "depth": 1,
 "info": {
 "info1": [1, 2, 3, 4],
 "info2": "."
    }
}
```

The header line is followed by a variable number of interval lines which are quite similar to the BED format:

```
chr start end orientation mods
```

The fields are tab-separated:

- The `chr` field is the name of the contig. Different intervals of the same molecule can be on different contigs.
- The `start` and `end` fields are the start and end positions of the interval (0-based, end-exclusive).
- The `orientation` field is the orientation of the interval (`+` or `-`). Different intervals of the same molecule can have different orientations.
- The `<mods>` field can be empty but the tab character preceding it is required. The `<mods>` is a list of comma separated base substitutions (no indels) local to the interval sequence represented in the current line. The substitutions are applied to the interval sequence before the strand is flipped (if the strand is `-`).

For example, consider the following FASTA contig and MDF entry:

```
FASTA:
>chr1
AGTCCCGTAA

MDF:
+m1 1
chr1 0 4 + 2C,3T
chr1 6 9 + 1G
```

Given the FASTA and MDF records, we can construct the sequence of the `m1` molecule.

- First we construct the sequence of the first interval: `(chr1, 0, 4) = AGTC`.
- We apply the modifications on positions 2 and 3: `AGTC -> AGCC -> AGCT`.
- Then we construct the second interval: `(chr1, 6, 9) = GTA`.
- We apply the modification on position 1: `GTA -> GGA`.
- Finally, we concatenate the two intervals to get the sequence of the `m1` molecule: `AGCTGGA`.

If the contig name is not in the provided reference FASTA but is nonetheless a valid nucleic sequence, TKSM will use the contig name as the contig sequence. Consider for example the following FASTA and MDF records:

```
FASTA:
>chr1
AGTC

MDF:
+m1 1
TT 0 2 +
chr1 0 4 +
```

Given the FASTA and MDF records, we can construct the sequence of the `m1` molecule.

- First we construct the sequence of the first interval. Since `TT` is not a reference name in the FASTA file, we treat it as a sequence itself: `("TT", 0, 2) = TT`.
- We construct the sequence of the second interval: `(chr1, 0, 4) = AGTC`.
- Finally, we concatenate the two intervals to get the sequence of the `m1` molecule: `TTAGTC`.

S1.2 Defining TKSM pipelines

To use Snakemake as a pipeline management software to run TKSM, we built a Snakemake script that takes as configuration a `YAML` file. The `YAML` file describes the order of modules to be used in a given simulation experiment as well as the relevant datasets used as models in the pipeline. Listing S1 presents an example for defining the Snakemake configuration file for TKSM simulation pipeline.

```yaml
TS_experiments:
    head_1:
        pipeline:
            - Tsb:
                params: "--molecule-count 200000"
                model: "MCF7-sgnex"
                mode: Xpr
            - Trc:
                params: ""
                model: "MCF7-sgnex"
            - plA: {}
    head_2:
        pipeline:
            - Tsb:
                params: "--molecule-count 100000"
                model: "N1"
                mode: Xpr_sc
            - Trc:
                params: ""
                model: "N1"
            - plA: {}
            - SCB: {}
            - Tag:
                params: "--format5 10"
    experiment_1:
        pipeline:
            - Mrg:
                sources: ["head_1", "head_2"]
            - PCR:
                params: "--cycles 10 -x T4 --molecule-count 1000000"
            - Flp: {}
            - Seq:
                params: "--skip-qual-compute"
                model: "nanopore2020"
samples:
    "MCF7-sgnex":
        fastq:
            - data/samples/MCF7-sgnex.fastq.gz
        ref: Homo_sapiens
    "N1":
        fastq:
            - data/samples/N1.fastq
        ref: Homo_sapiens
refs:
    Homo_sapiens:
        cDNA: data/refs/Homo_sapiens.cdna.fa
        DNA: data/refs/Homo_sapiens.dna.fa
        GTF: data/refs/Homo_sapiens.gtf
barcodes:
    10x_bc: data/refs/3M-february-2018.txt.gz
```

Listing S1: An example for a TKSM Snakemake configuration file with a simulation pipeline that uses as input the outputs of two other simulation pipelines.

S1.3 Abundance estimation module

The abundance estimation utility takes as input a PAF file containing the alignment information of the long-reads of a real transcriptomic dataset to the transcriptome. We use Minimap2 [Li, 2018] to perform this transcriptomic mapping. Similarly to Trans-Nanosim [Hafezqorani et al., 2020], it computes the estimated abundance of different transcripts using an Expectation-Maximization (EM) algorithm implementation by Simpson (`https://github.com/jts/nanopore-rna-analysis`) which estimates transcript abundance from multi-mapping long-reads. This utility generates the transcript counts normalized by the total throughput and divided by one million (transcript per million or TPM).

Single cell expression. We augmented this abundance estimation method so that it can also accept cellular barcode tags, generated by tools such as scTagger [Ebrahimi et al., 2022] or FLAMES [Tian et al., 2021]. This allows the generation of single-cell resolution transcript expression profiles, and thus generate simulated single-cell datasets. To the best of our knowledge, TKSM is the only simulator that can generate single-cell transcriptomics long-read datasets. To do that, we maintain a separate count for each pair of cellular barcode and transcript. We then feed these counts to the standard EM algorithm for abundance estimation.

S1.4 Transcribing module

The Transcribing module takes as input a gene annotation file (GTF) describing the genomic intervals corresponding to the transcripts and the transcript abundance estimation profile generated by TKSM Abundance estimation utility and generates a set of molecules from which sequencing reads will be simulated. The Transcribing module is an entry-point module and thus it does not take an MDF file as input and it generates an MDF file as output. Given a user-defined parameter N, the module samples N transcripts from the GTF file according to their abundance frequencies and outputs them in MDF format. Any cellular barcode information present in the abundance file for a given transcript is recorded in the output MDF.

S1.4.1 Gene fusion submodule

The Transcribing module can optionally generate gene fusion transcripts, for gene fusions induced by genomic structural variants. The user can specify genomic breakpoints of fusion-inducing structural variations (e.g. deletion, inversion or translocation); otherwise, they are randomly chosen. Then, gene fusion transcripts are generated randomly from the transcripts of the involved genes and their expression. As the MDF format enables the representation of various combinations of genomic intervals, irrespective of their chromosome or strand, and as downstream modules are indifferent to the molecular content, fusion transcripts will be handled like any other molecule in any subsequent TKSM modules.

Fusion simulation is implemented as a sub-module to the transcribe module and activated when any fusion-related parameter is passed to the transcriber. This submodule can be summarized in three steps; characterization, configuration and modification.

The characterization step aims to define a list of genomic events that will induce gene fusion transcripts. Genomic events in this context are defined by two breakpoints (head and tail where fusion transcripts pass head before the tail) with contig, position and orientation and event rate which can be 1 for homozygous, 0.5 for heterozygous events and any value between 1 and 0 to indicate fusion population in somatic samples. These events are indexed for efficient access in the latter steps. The result of this step is a list of pairs of breakpoints, each pair being composed of one breakpoint within each of the tail and head genes of the fusion. While biologically it is possible to have overlapping events (resulting in complex fusions), for the sake of clarity TKSM only simulates non-overlapping events.

The configuration step takes the gene annotation (GTF) and transcript abundances passed to the transcriber and the genomic events generated in the prior step. For each genomic event, transcripts overlapping with the breakpoints are gathered. Fusion transcripts are determined by the set of transcript pairs generated by the Cartesian product of the head-overlapping and tail-overlapping transcripts (Figure S1). Total fusion expression is set by the sum of head transcript expression. This total expression is distributed across the fusion transcripts by following the Cartesian product of head-overlapping and tail-overlapping transcript expressions. Fusion transcript sequences are determined by concatenating the head exons before and tail exons after the breakpoints. Equivalent fusion transcripts are merged and their expression values are accumulated.

Finally, the modification step adds the generated fusion transcript annotations and abundances to the simulation. Then, it modifies the expressions of the transcripts overlapping with the generated fusion

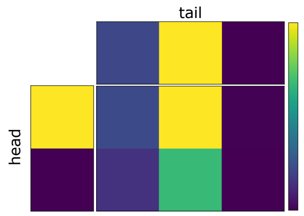

Figure S1: Cartesian product to decide fusion transcript expression. Each cell represents a transcript coloured for its expression values (blue to yellow). In this example, two genes with 2 and 3 transcripts are fused resulting in six fusion transcripts.

Algorithm S1 TKSM algorithm for generating random gene fusion events.

Input: Genes, FusionCount
$Genes \leftarrow shuffled(Genes)$
$G \leftarrow Genes[: 2 \times FusionCount]$ # *Take first 2 * FusionCount genes on shuffled Gene list*
$G \leftarrow sorted(G)$ #*by the genomic positions*
$fusion\_events \leftarrow list()$
for each consecutive (g_1,g_2) in G **do**
  $b_1 \leftarrow$ randomInt(g_1.start, g_1.end)
  $b_2 \leftarrow$ randomInt(g_2.start, g_2.end)
  fusion_events.add(FusionEvent(g_1,g_2, b_1, b_2))
end for

Algorithm S2 TKSM algorithm for generating fusion isoforms.

Transcripts, fusion_events
for each event in $fusion\_events$ **do**
  $t\_head \leftarrow Transcripts.overlaps(event.g_1)$
  $t\_tail \leftarrow Transcripts.overlaps(event.g_2)$
  $head\_count \leftarrow sum(t\_head.counts)$
  $tail\_count \leftarrow sum(t\_tail.counts)$
  $fusion\_isoforms \leftarrow list()$
  for each t_1 in t_head **do**
    for each t_2 in t_tail **do**
      $fusion\_count \leftarrow head\_count \times \frac{t_1.count}{head\_count} \times \frac{t_2.count}{tail\_count}$
      $iso_1 \leftarrow t_1.truncate(t_1.start, event.b_1)$
      $iso_2 \leftarrow t_2.truncate(t_2.start, event.b_2)$
      $fusion\_isoforms.add(concat(iso_1, iso_2), fusion\_count)$
    end for
  end for
end for

---

events (on breakpoints or anything in between for deletions), reducing it with respect to the rate of the event.

S1.5 Single-cell barcoding module

This module adds single-cell barcode sequences to the molecules using data generated by the Transcribing module. If the expression data passed to the Transcribing module was generated in single-cell expression mode, the Transcribing module will add the cellular barcode information of each molecule in a special tag (e.g. `CB:ACTACGAAGAAACCAT`) in its MDF output. These cellular barcode tags are mainly used by the Single-cell Barcoding module to add the barcode sequence to their respective molecules.

S1.6 Tagging module

The Tagging module inserts custom sequences into the simulated molecules. This enables the user to add any combination of primers and/or UMI tags to the simulated molecules. The Tagging module is flexible and accepts IUPAC formatted strings of nucleotide codes to be appended at either the $5'$ or $3'$

ends of the molecules. For example, `tksm tagging -3 AYNN` will append at the start of each molecule a random tag that begins with `A`, followed by `C` or `T`, followed by 2 random nucleotides.

S1.7 Filtering module

The Filtering module takes a series of conditions on the molecule records and filters any molecules that fail one or more of these conditions. The module supports conditions on the length of the molecule, overlaps with genomic loci or chromosomes, and the presence of specific tags (e.g. cellular barcode). This module is useful for creating different pipelines for the molecules that pass the filter and those that fail it.

S1.8 PCR module

The PCR module duplicates the input material simulating the PCR process in a manner similar to work done in Calib [Orabi et al., 2019] and Minnow [Sarkar et al., 2019]. The PCR module takes as parameters the number of PCR cycles, c, the error rate per duplicated base, e, and the PCR efficiency, $f \in [0, 1]$. Additionally, the PCR module takes the desired number of molecules, N, to be selected from the exponentially many molecules that will be present at the end of the PCR process. Conceptually, in each PCR cycle, the module randomly selects a set of molecules to duplicate equal to the number of input molecules multiplied by f. It then inserts random substitution errors to the duplicated molecules equal to their total length multiplied by e. It then proceeds to the next cycle using the old and new sets of molecules as input. Finally, from the exponentially many molecules created, the modules randomly output N of them.

The parameter N is generally a small fraction of the number of molecules present at the end of the PCR process, which is equal to $M \times (1 + f)^c$ where M is the number of input molecules. Therefore, creating all the PCR molecules at the same time would lead to a huge memory footprint. Rather, TKSM processes each molecule independently by creating a truncated duplication tree for it. Initially, the tree has a single node representing the original molecule. In each PCR cycle, the module decides randomly (with a success rate of f) for each node in the tree if this node should be expanded into a subtree. If the expansion test is successful, TKSM adds a new child to the node, associated with a newly duplicated molecule obtained from its parent by simulating a number of random mutations equal to the molecule length multiplied by e. Then TKSM decides whether to capture (i.e. output) this newly created molecule randomly with a success rate equal to the probability of capturing a molecule in the output: $N/\big(M \times (1 + f)^c\big)$.

S1.9 polyA tails addition module

The polyA module appends a polyA tail to the molecules in its input MDF following a normal distribution of the length of the tails with a specified mean and standard deviation. To estimate these parameters from a real dataset, we use a simple script described by Orabi et al. [2023] to detect the length of the polyA tails of the reads of the real dataset.

S1.10 Strand flipping module

This module takes an MDF and randomly reverses the order of the intervals of the molecules in the MDF according to a user-defined probability p. If a molecule is reversed, the module then adds a tag to its intervals indicating that their sequence is reversed complemented. The module is useful for simulating sequencing protocols that are not strand-specific.

S1.11 Gluing module

The Gluing module takes an MDF and a user-specified probability p. It then processes each molecule, and with probability $1 - p$ it outputs the molecule with no modification and with probability p it prepends the molecule to the next molecule. The Gluing module aims to simulate the behaviour of the ONT signal mis-segmentation process in which sometimes the ONT software fails to segment the signal of two consecutive molecules and as a result outputs their sequences as a single read.

S1.12 Shuffling module

This module takes an MDF and outputs its molecules in random order. To allow for reduced output latency and memory consumption, the module buffers the input into a dynamically allocated array with a maximum size of N (user-defined with a default of $N = \infty$). Once the buffer array is full, the module will randomly output one of its molecules and replace it with a new incoming molecule. Once the input is exhausted, the buffer array is shuffled in place and its molecules are outputted. The smaller N is, the more localized the shuffling will be. The randomized shuffling of the molecules enables the user to generate a random subsample from an MDF and is necessary for modules that assume a random order of the molecules such as the Gluing module.

S1.13 Truncating module

This module simulates the process by which only a portion of the molecule is sequenced due to truncation. Such sequence truncation is caused by library preparation artifacts or, specifically in the case of ONT sequencing, by early stopping of the sequencing due to pore blocking [Soneson et al., 2019, Amarasinghe et al., 2020]. To simulate the truncation of transcriptomic molecules, we use a two-dimensional kernel density estimation (KDE) model to decide the truncation length with respect to the transcript length, similar to Trans-Nanosim [Hafezqorani et al., 2020]. We also implement a simple binning-based approach to distribute the truncation length on the 3' and 5' ends of the molecule.

TKSM models the truncation process as a function $f(x) = y$ where the truncation length of the molecule, y, is a function of the full length of the molecule, x. When estimating f, TKSM uses the mapping of real transcriptomic reads to the set of reference transcripts. In particular, the length of the transcript is taken as x while the total truncation ($TLEN - TEND + TSTART$) of the read alignment is taken as y. In TKSM, we use the scikit-learn [Pedregosa et al., 2011] implementation of the KDE. In scikit-learn implementation, we can sample the KDE model to get (x, y) pairs. However, for the purpose of the truncation module, we need to sample the KDE for a y (truncation length) value given an x value (transcript length).

To enable sampling the KDE model for y given x, we compute the likelihood of a large predefined set of (x, y) pairs. This set of pairs is defined by a cross-product of a set of x values, X, and a set of y values, Y. By default, we set $X = 0 : 100 : 10000$ and $Y = 0 : 100 : 10000$. We then use the `score_samples` function of scikit-learn on each (x, y) point in $X \times Y$ to get the likelihood of each point. When given an x value of a specific molecule, we find the points x' and x'' on X that are closest to x. We then define $f(x)$ to be the mean of $f(x')$ and $f(x'')$ weighted by their distance from x. This process is detailed in Algorithm S3 and illustrated in Figure S2.

Algorithm S3 TKSM truncation KDE sampling method.

Inputs:
x: input molecule length
X, Y: Predefined lattice points
$\mathcal{L}$: Precomputed vector of likelihood functions
$x' \leftarrow lower\_bound(X, x)$
$x'' \leftarrow upper\_bound(X, x)$
$\mathcal{C}_1 \leftarrow cumsum(\mathcal{L}[x'])$
$\mathcal{C}_2 \leftarrow cumsum(\mathcal{L}[x''])$
$u \leftarrow uniform\_random(0, 1)$
$i_1 \leftarrow lower\_bound(\mathcal{C}_1, u)$
$i_2 \leftarrow lower\_bound(\mathcal{C}_2, u)$
$t_1 \leftarrow Y[i_1]$
$t_2 \leftarrow Y[i_2]$
$w_1 = |x - x'|$
$w_2 = |x - x''|$
return $weighted\_mean(t_1, t_2, w_1, w_2)$

Since the KDE model building procedure is computationally intensive, we implemented it in TKSM as a preprocessing utility, which computes and saves the lattice likelihood values. The pre-built models can then be used by the Truncating module.

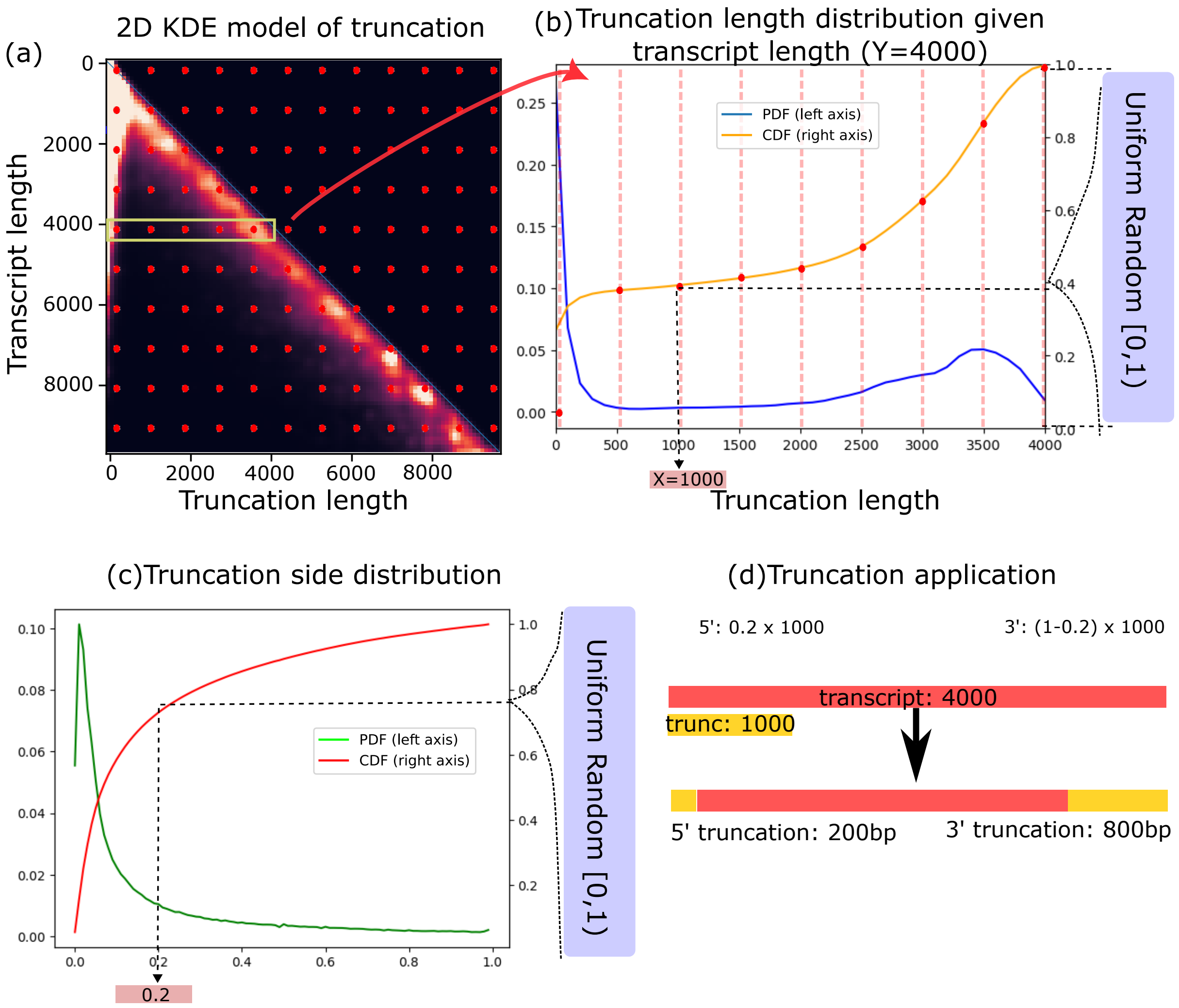

Figure S2: KDE Truncation method. A) Grid of values (500bp apart in this example, but is more frequent in practice) are selected and likelihoods of each (x,y) pair on the KDE model are computed. To generate truncation lengths for specific transcript lengths, TKSM takes a slice of likelihood values of the appropriate size. After normalization, these likelihoods are used as an estimation of a probability mass function (PMF) and cumulative distribution function (CDF). B) Then TKSM converts uniform random values to random values on this distribution by finding lower bounds on CDF values using binary search (weighted average strategy shown in the algorithm S3 is omitted in this figure for clarity). (C) Similar to the truncation length decision process, TKSM decides the ratio of 5' and 3' truncation by sampling from a CDF derived from the distribution of truncation lengths of the 3' and 5' ends in the real datasets. D) Truncation lengths generated in B are distributed to 5' and 3' ends according to the ratio generated in C and applied to the input molecules.

S1.14 Merging module

The Merging module is a simple module that concatenates the MDF outputs of one or more other TKSM modules into a single MDF file. This module is another entry-point module alongside the Transcribing module. The main use of this module is to enable simulation pipelines that take as input the output of one or more simulation pipelines; for example, the user may use this module to build a mixed-sample simulation dataset by merging the MDF output of multiple pipelines that use the Transcribing modules on different samples.

In the piped version of TKSM, the Merging module is slightly more complicated than a simple concatenation operation. This is because concatenating files linearly and in-order can result in a deadlock when the input and output of the modules are piped. To avoid such deadlock, we implemented a multi-threaded version of the Merging module that assigned a CPU thread to each file, concurrently consuming the input files.

S1.15 Tail-noise module

The Tail-noise module simulates the low-quality sequences at the end of the reads often generated by the long read sequencing technologies as an artifact. Given a length distribution and an alphabet, it adds randomly generated sequences to each molecule. The rate of each letter in the alphabet can be controlled by repeating it in the alphabet string. For example, $\Sigma = AAAGTC$ will produce a noise sequence with 50% As. Alternatively, two create a palindromic noise effect, the tail-noise module can attach mirrored and erroneous copy of the molecule's tail. The error profile of these mirrored sequences can be controlled with a user-defined error rate.

S1.16 Sequencing module

The Sequencing module takes an MDF and the genome reference file(s) of the simulated sample and generates sequencing reads of the molecules in the MDF. The module can generate perfect reads (i.e. with no base-level errors) or erroneous reads, under a given error model. To generate erroneous reads we integrated some functions from the Badread [Wick, 2019] long-read simulator into a multi-threaded implementation in the Sequencing module. We also used Badread's method of building a k-mer substitution error and quality score model. As part of TKSM Snakemake, the user may specify to train Badread models on the given real samples or to use pre-trained Badread models. This allows TKSM to be easily applied to new or future long-read sequencing chemistries.

S2 Supplementary Results

Our goal in this section is to demonstrate that TKSM is capable of producing simulated sequencing data that has realistic characteristics in terms of its biological features (e.g. isoform expression) and technological artifacts (e.g. sequencing error). To assess these characteristics, we compare the simulated datasets to the input real dataset they were based on (MCF7 for bulk RNA-seq and N1 for scRNA-seq). Additionally, we want to assess TKSM memory and time usage while it generates these sequencing data.

S2.1 `YAML` definitions of the experiments

```yaml
TS_experiments:
    bulk_RNA:
        pipeline:
            - Tsb:
                params: "--molecule-count 1000000"
                model: "MCF7-sgnex"
                mode: Xpr
            - Trc:
                params: ""
                model: "MCF7-sgnex"
            - plA:
                params: "--normal=15,7.5"
            - Flp:
                params: "-p 0.5"
            - Tag:
                params: "--format5 AATGTACTTCGTTCAGTTACGTATTGCT --format3 GCAATACGTAACTGAACGAAGT"
            - Seq:
                params: "--skip-qual-compute"
                model: "MCF7-sgnex"
```

Listing S2: TKSM Snakemake configuration file for the bulk RNA-seq simulation experiment.

```
TS_experiments:
    single_cell_head:
        pipeline:
            - Tsb:
                params: "--molecule-count 1000000"
                model: "N1"
                mode: Xpr_sc
            - Trc:
                params: ""
                model: "N1"
    single_cell_p1:
        pipeline:
            - Mrg:
                sources: ["single_cell_head",]
            - Flt:
                params: "-c \"info CB\""
            - plA:
                params: "--normal=15,7.5"
            - Tag:
                params: "--format3 10"
            - SCB:
                params: ""
            - Tag:
                params: "--format3 AGATCGGAAGAGCGTCGTGTAG"
    single_cell_p2:
        pipeline:
            - Mrg:
                sources: ["single_cell_head",]
            - Flt:
                params: "-c \"info CB\" --negate"
    single_cell:
        pipeline:
            - Mrg:
                sources: ["single_cell_p1", "single_cell_p2"]
            - PCR:
                params: "--cycles 5 --molecule-count 5000000"
            - Flp:
                params: "-p 0.5"
            - Tag:
                params: "--format5 AATGTACTTCGTTCAGTTACGTATTGCT --format3 GCAATACGTAACTGAACGAAGT"
            - Seq:
                params: "--skip-qual-compute"
                model: "N1"
```

Listing S3: TKSM Snakemake configuration file for the single-cell RNA-seq simulation experiment.

```
TS_experiments:
    gene_fusion_RNA:
        pipeline:
            - Tsb:
                params: "--molecule-count 1000000 --fusion-count 100"
                model: "MCF7-sgnex"
                mode: Xpr
            - Trc:
                params: ""
                model: "MCF7-sgnex"
            - plA:
                params: "--normal=15,7.5"
            - PCR:
                params: "--cycles 5 --molecule-count 5000000"
            - Flp:
                params: "-p 0.5"
            - Tag:
                params: "--format5 AATGTACTTCGTTCAGTTACGTATTGCT --format3 GCAATACGTAACTGAACGAAGT"
            - Seq:
                params: "--skip-qual-compute"
                model: "MCF7-sgnex"
```

Listing S4: TKSM Snakemake configuration file for the bulk RNA-seq simulation experiment with 100 random gene fusion events added (option `--fusion-count 100` of the `Tsb` module).

S2.2 Transcript expression profiling

We used LIQA [Hu et al., 2021] to compute the expression profiles of the different simulated and real bulk RNA-seq datasets. We additionally generated transcript expression profiles using Minimap2, trans-Nanosim, and TKSM. For Minimap2, we used the number of primary alignments of the long-reads to the transcripts as transcript counts. For trans-Nanosim and TKSM, we use the `read_analysis.py quantify` and `tksm abundance` commands, respectively, to generate transcript counts. We then normalize all the generated counts to be transcript-per-million (TPM) counts by dividing each transcript count by the total expression and multiplying by one million. For the scRNA-seq dataset, we used TKSM single-cell mode of the abundance estimation utility. We then combine the TPM counts of each gene since the number of unique single-cell barcode and transcript pairs is very large compared to the number of generated reads. The TPM counts using these different methods on the bulk RNA-seq datasets are plotted in Figures S3 and S4.

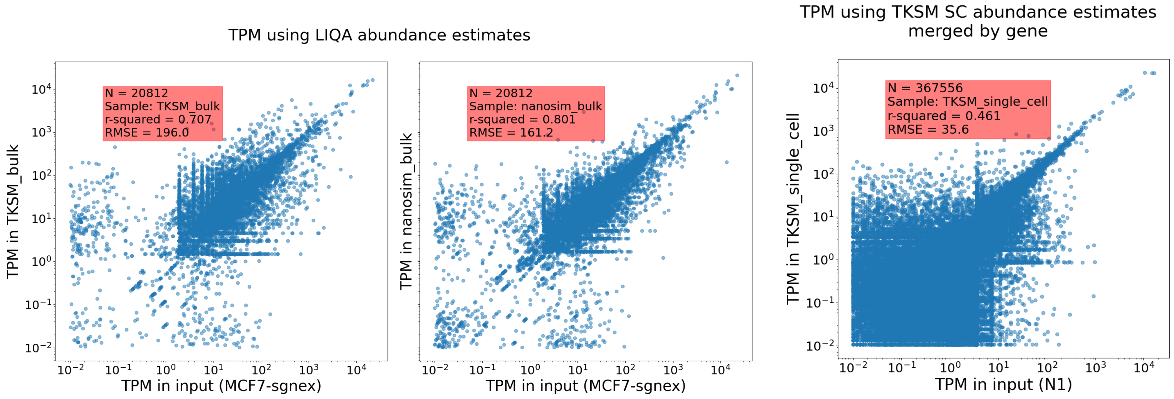

Figure S3: On the left, transcript expression (TPM) of bulk RNA-seq datasets is computed by LIQA. Each point represents a single transcript and its expression. On the right, hybrid scRNA-seq using TKSM single-cell transcript expression utility. Each point represents the expression sum of the transcripts of the same gene and cellular barcode.

On the bulk RNA-seq datasets, TKSM and Trans-Nanosim have high concordance with the input real dataset with correlation coefficients of 0.71 and 0.71, respectively. Both tools have small root mean square error (RMSE) rates of 196 and 161, respectively. On the scRNA-seq dataset, TKSM has a lower correlation coefficient of 0.46 compared to its performance on the RNA-seq dataset. However, a reduction in the correlation coefficient for the scRNA-seq dataset is expected since it includes over 367K gene/barcode data points compared and thus has a lot more opportunity for variation from the input counts.

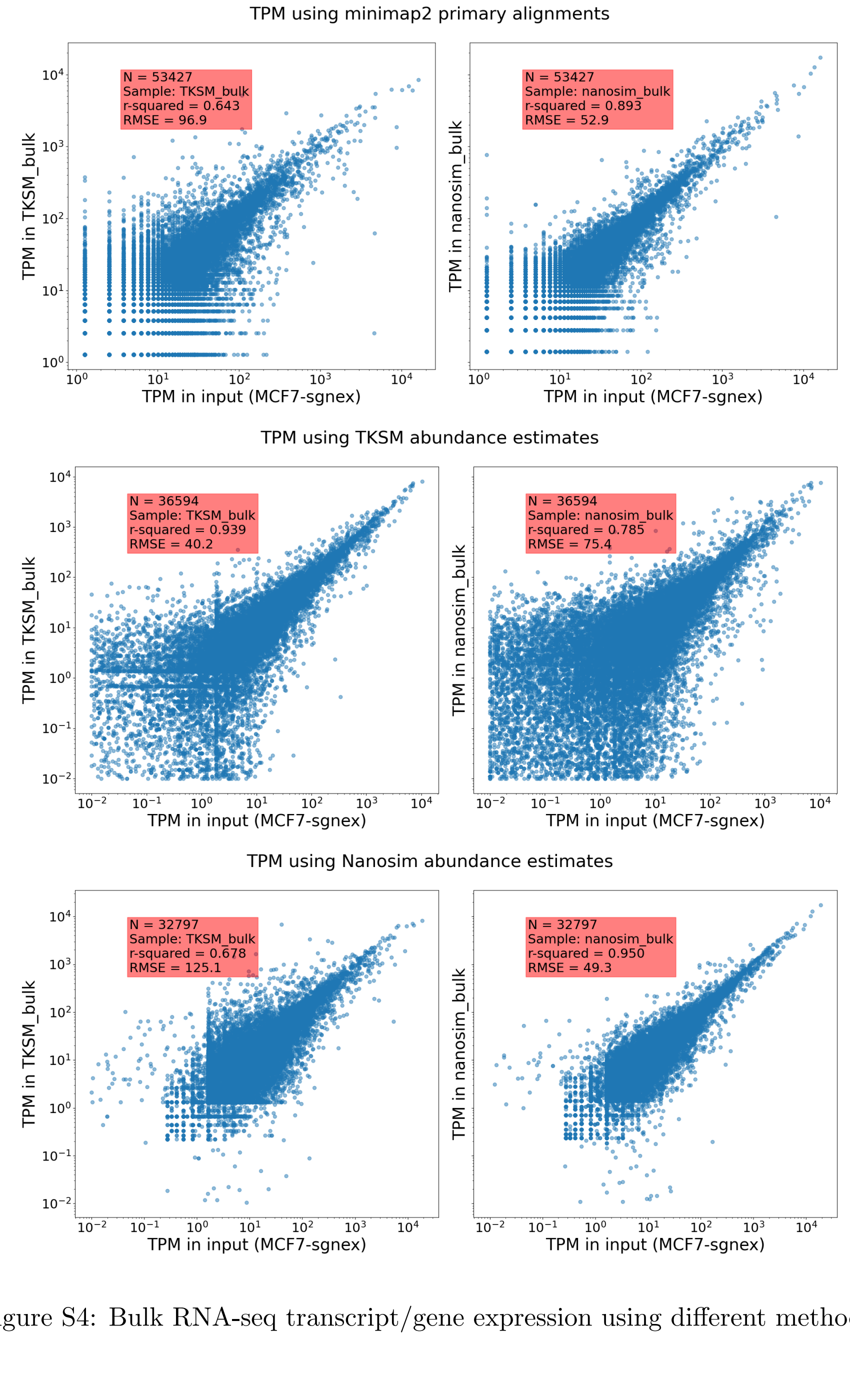

Figure S4: Bulk RNA-seq transcript/gene expression using different methods.

S2.3 Truncation

To measure the truncation level of the sequencing pipeline, we mapped the long-reads of the different datasets to the transcriptome using Minimap2. For each primary alignment, we compute the read mapping length ($target\_end - target\_start$) which we use as a proxy for the post-truncation sequencing length. The mapping lengths are plotted as bar graphs in Figure S5. Both Trans-Nanosim and TKSM generate very similar read length distributions compared to the input datasets. We also plot the distribution of the truncation length on the molecules' start vs. end in Figure S6. TKSM results in a much more realistic distribution of the truncation within the simulated molecules. On the other hand, Trans-Nanosim has an almost uniform distribution of the truncation length.

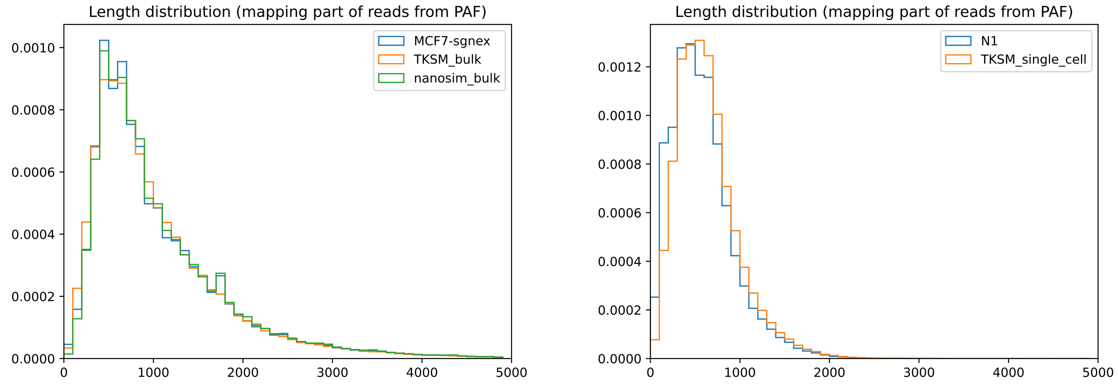

Figure S5: Mapped length of the reads vs the length of the transcript of their primary alignment using Minimap2. On the left, bulk RNA-seq datasets are plotted. On the right, scRNA-seq datasets are plotted.

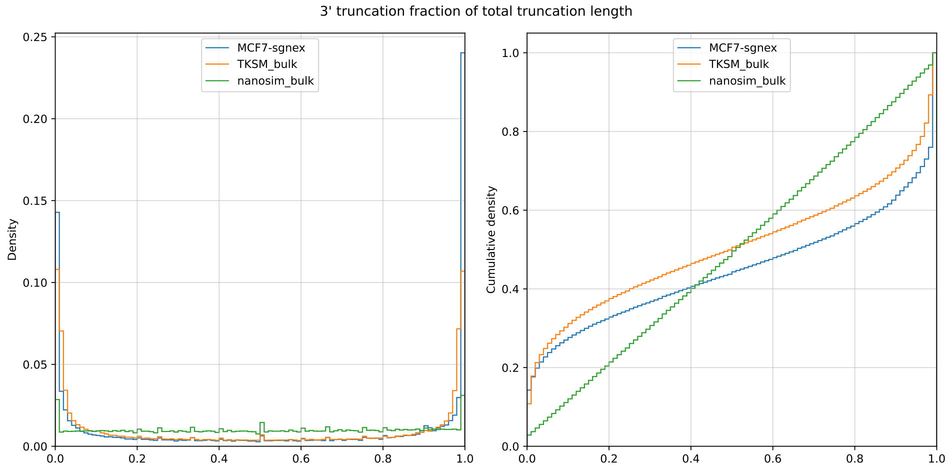

Figure S6: $3'$ share of the total truncation length on the bulk RNA-seq datasets. Total truncation length is calculated from the PAF (cDNA mappings): $total\_truncation = t_{start} + t_{length} - t_{end}$. If the alignment strand is "+", $3'$ share is $\frac{t_{length} - t_{end}}{total\_truncation}$, otherwise it is $\frac{t_{start}}{total\_truncation}$. Left plot: probability density distribution. Right plot: Cumulative sum of the left.

S2.4 PolyA tail lengths

To compute the length of the observed polyA tails on the long-reads, we use a simple polyA detection method described by Orabi et al. [2023]. The distribution of the polyA tails and lengths is plotted in Figure S7. The real N1 scRNA-seq dataset seems to have a bi-modal distribution (polyA tails < 10bp and ~ 25bp) which are not both captured by the TKSM simulated dataset. However, the distribution observed for the TKSM dataset is closer to the real data distribution compared to Trans-Nanosim.

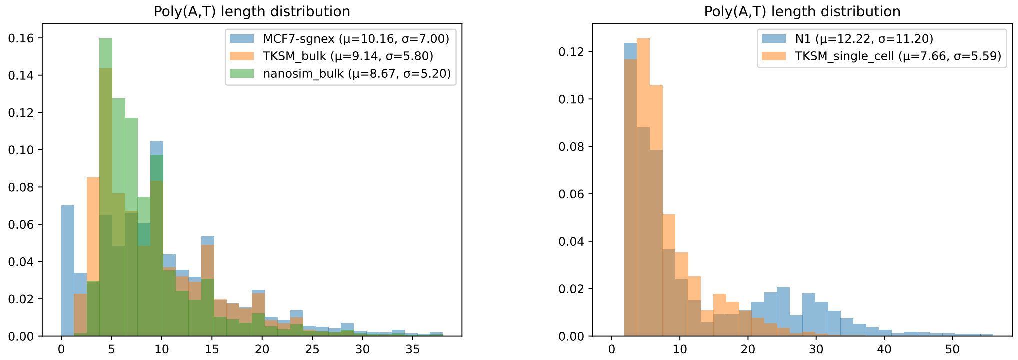

Figure S7: PolyA length of the reads as computed by TKSM. On the left, are lengths in the bulk RNA-seq datasets and, on the right, are lengths in the scRNA-seq datasets.

S2.5 Base-level sequencing errors

To assess the base-level sequencing error profile of the datasets, we aligned the reads to the transcriptome using Minimap2 with `-c` flag to generate the alignment CIGAR strings. We then compute, as a percent of the mapped read length, the match, substitution, insertion, and deletion rates. The distributions of the sequencing errors are plotted in Figure S8 both for the bulk RNA-seq and scRNA-seq datasets.

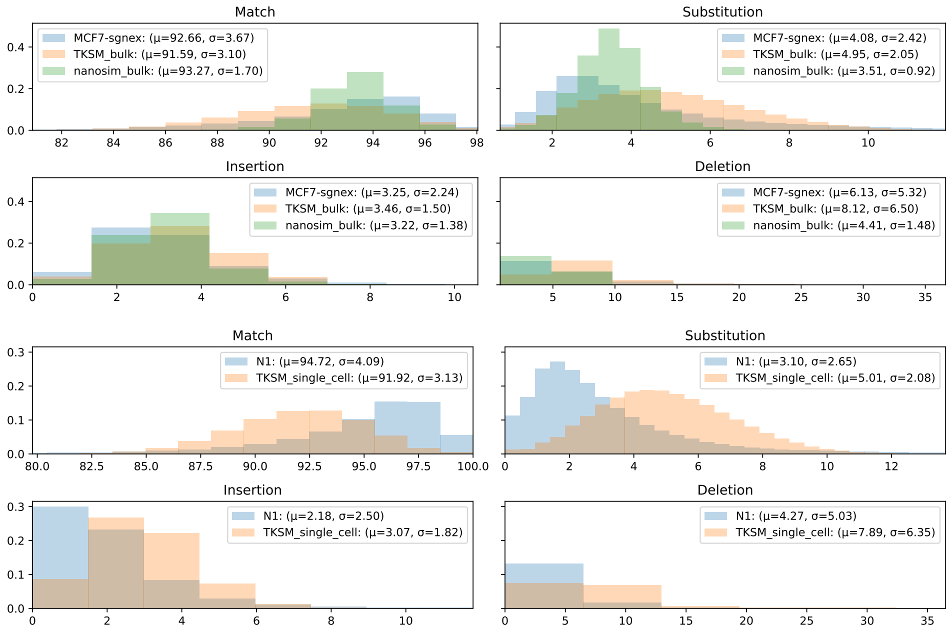

Figure S8: Substitution, insertion, and deletion base-level error rates using the CIGAR strings of Minimap2 mapped reads of the bulk RNA-seq (top two rows) and scRNA-seq datasets (bottom two rows).

S2.6 Single-cell barcode profile

To assess the distribution of edit errors on the cellular barcodes present on the long-reads, we performed pair-wise alignment of each whitelist cellular barcode to each long-read using Edlib [Šošić and Šikić, 2017] Python package. Instead of aligning to the whole long-read, we only considered the ranges $[25, 75)$ and $[-75, -25)$ of the long-reads to reduce the running time. The cellular barcodes whitelist was generated by running scTagger [Ebrahimi et al., 2022] on the full long-read dataset. We considered each cellular barcode and its reverse complement as independent barcodes. Only the alignments minimizing the edit distance, d, are kept from all the computed pair-wise alignments. If only one such alignment exists, then we consider it a unique alignment. Otherwise, we consider the alignments to be ambiguous. The TKSM generated dataset has a realistic distribution of cellular barcode matching in terms of the ambiguity of assigning each long-read to a barcode and the number of errors detected on the barcode as shown in Figure S9.

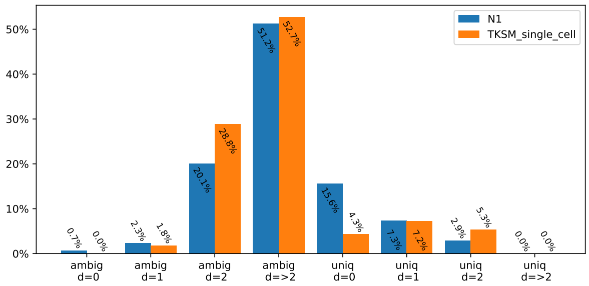

Figure S9: Brute-force detection of the cellular barcodes on a random sample of 50,000 long-reads from each dataset.

We also wanted to assess the distribution of where the Illumina short-read adapter is located on the long-reads. To compute these loci, we ran scTagger on the scRNA-seq long-read datasets. We observe that the simulated dataset generated by TKSM has a similar distribution to the real dataset in terms of the loci of the SR adapter on the LRs and in terms of the number of LRs that scTagger is unable to detect a SR adapter on. This is demonstrated in Figure S10.

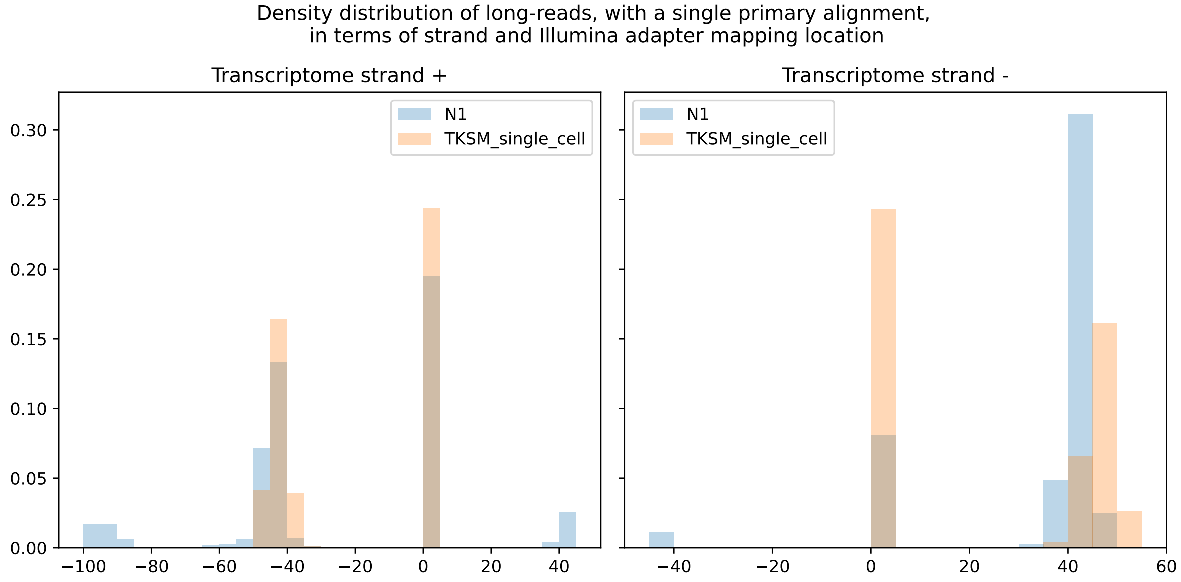

Figure S10: Distribution of the detected Illumina short-read adapter on the long-reads separated by the transcript strand to which the long-read maps. The x-axis represents the match location of the Illumina adapter on the long-read. Negative x-axis values represent the match location from the end of the read for Illumina adapters that map on the negative strand of the long-read. The 0 point on the x-axis represents long-reads that have no Illumina adapter detected on them.

S2.7 Gene fusion experiment

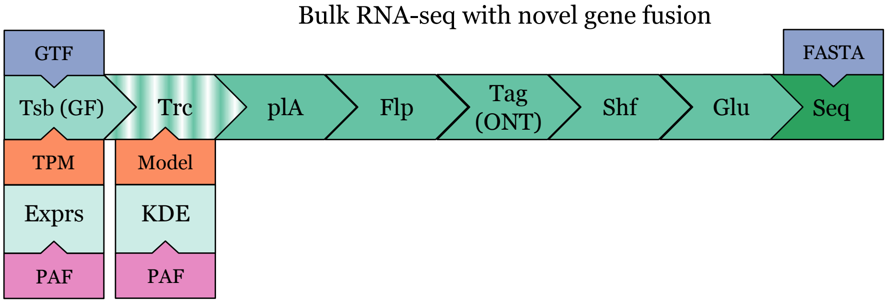

Figure S11: A TKSM RNA-seq pipeline that creates novel gene fusion events using the Transcribing module. The Glu module creates mis-segmentation errors resulting in chimeric reads. We also simulated datasets with and without truncation to evaluate the effect of truncation on the accuracy of gene fusion discovery.

Using TKSM, we simulated 50 random gene fusions, i.e., gene fusions generated from randomly selected gene pairs located on the same chromosome. We simulated datasets with and without truncation and with several mis-segmentation rates (0%, 1%, 2%, 4%, and 8%). Figure S11 illustrates the TKSM pipeline we designed for this gene fusion experiment. To test these datasets, we used gene fusion discovery tools Genion [Karaoglanoglu et al., 2022] and LongGF [Liu et al., 2020].

We observed that truncation negatively affects gene fusion detection. Note that our simulation pipeline is not strand-specific, i.e., molecules are sequenced from either end with equal probability. In this experiment, the inclusion of the truncation raised the number of undetectable fusions by either tool from 7 to 18 (Figure S12). While LongGF was resilient to the mis-segmentation, we observed a drop in the recall rate of the Genion especially when truncation was introduced to the dataset (Table S1). Additionally, when the mis-segmentation rate increases from 0% to 8%, the number of candidate gene fusions detected by Genion, but subsequently discarded by one of its filters, increases $\sim 5\times$. These findings show that TKSM can generate gene fusion targets as well as un-segmentation errors that have direct effect on the running of gene fusion detection tools.

Truncation	Glue rate	Tool	#	TP	FP	FN	F1	Truncation	Glue rate	Tool	#	TP	FP	FN	F1
FALSE	0%	LongGF	39	38	1	12	0.85	TRUE	0%	LongGF	34	30	4	20	0.71
		Genion	44	42	2	8	0.89			Genion	34	31	3	19	0.74
	1%	LongGF	43	42	1	8	0.9		1%	LongGF	33	30	3	20	0.72
		Genion	43	41	2	9	0.88			Genion	32	30	2	20	0.73
	2%	LongGF	43	42	1	8	0.9		2%	LongGF	33	30	3	20	0.72
		Genion	42	41	1	9	0.89			Genion	31	30	1	20	0.74
	4%	LongGF	44	42	2	8	0.89		4%	LongGF	31	29	2	21	0.72
		Genion	41	40	1	10	0.88			Genion	29	27	2	23	0.68
	8%	LongGF	42	41	1	9	0.89		8%	LongGF	31	29	2	21	0.72
		Genion	41	40	1	10	0.88			Genion	23	23	0	27	0.63

Table S1: Gene fusion detection results in the output of TKSM. For all eight experiments, the same 50 gene fusions were simulated.

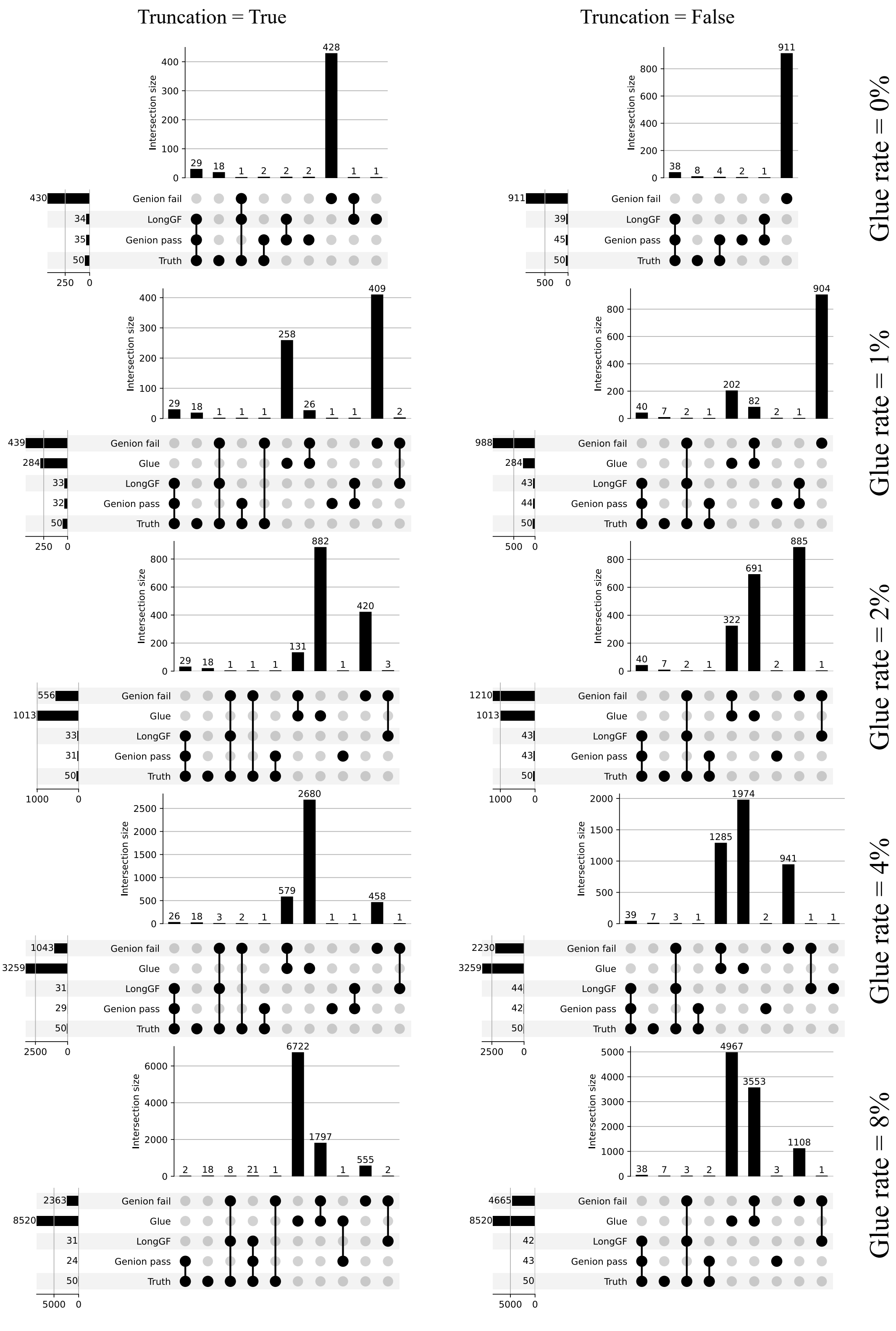

Figure S12: UpSet plots of the gene fusions simulated by TKSM or detected by Genion or LongGF. Note that UpSet plots are essentially Venn diagrams represented using bar graphs. For Genion, we report both the gene fusion detected by the tool (Genion pass) and those detected by Genion initially but later filtered by one of its filters (Genion fail). For the gene fusions simulated by TKSM, we separate between true gene fusions generated by the Transcribing module (Truth) and false gene fusions that are generated by the Gluing module when it glues molecules of different genes (Glue). Note that for false gene fusions generated by the Gluing module, we require at least three molecules supporting each gene fusion to consider it in these plots.

S2.8 Time and memory use

We used GNU Time to monitor the memory and CPU use of the tested tools. The detailed results for the different steps of all these experiments are presented in Tables S2 and S3. For both tools, we separate the preprocessing steps (e.g. mapping, sequence error profiling, cell barcode detection) from the core steps (e.g. molecule generation, sequencing). For TKSM, we run its core processes twice: once in regular (i.e. blocking) mode and once in piped mode.

Table S2: Time and memory use for bulk RNA-seq simulation experiments. Values reported by GNU Time (v1.9). All tools were allowed to run with up to 32 CPUs. For regular (i.e. blocking) TKSM runs, reported real and user times are the sum of individual processes and memory is the maximum memory use by any individual process. For piped TKSM runs, the reported time and memory values are captured by GNU Time for the whole pipeline treated as a single process.

Tool	Process	Real time (min)	User time (min)	Memory (GB)	CPUs
Nanosim (preprocess)	`NS_analysis`	26.4	538.1	36.2	32
	`NS_quantify`	19.8	65.4	6.8	32
	**Total**	**46.2**	**603.6**	**36.2**	**32**
Nanosim (simulate)	**Total**	**8.7**	**224.0**	**0.8**	**32**
TKSM (preprocess)	`minimap2`	6.7	169.1	9.4	32
	`abundance`	1.5	1.4	3.8	1
	`model_truncation`	0.4	3.0	0.2	32
	`minimap2 (badread)`	2.2	54.9	5.8	32
	`badread_error_model`	1.8	1.7	4.9	1
	`badread_qscore_model`	7.5	7.4	4.9	1
	**Total**	**28.9**	**461.5**	**9.4**	**32**
TKSM (core)	`transcribe`	1.3	1.2	3.5	1
	`truncate`	0.2	0.2	0.0	1
	`polyA`	0.3	0.3	0.0	1
	`flip`	0.3	0.3	0.0	1
	`tag`	0.4	0.4	0.0	1
	`sequence`	3.1	83.7	3.3	32
	**Total**	**5.5**	**86.0**	**3.5**	**32**
TKSM (core;piped)	`transcribe`	3.9	1.1	3.5	1
	`truncate`	3.9	0.4	0.0	1
	`polyA`	3.9	0.5	0.0	1
	`flip`	3.9	0.5	0.0	1
	`tag`	3.9	0.6	0.0	1
	`sequence`	3.9	87.1	3.2	32
	**Total**	**4.0**	**90.1**	**3.5**	**32**

As we observe in Tables S2, TKSM finishes its preprocessing and core processing in, respectively, 36% and 37% less time than Trans-Nanosim despite generating the same amount of data and running similar pipelines. For the core processes, TKSM runs in 27% or 25% less time, respectively, on bulk RNA-seq and scRNA-seq datasets, when it runs in piped mode compared to its regular mode and without any increase in memory usage.

Table S3: Time and memory use for hybrid RNA-seq simulation experiments. Values reported by GNU Time (v1.9). All tools were allowed to run with up to 32 CPUs. For regular (i.e. blocking) TKSM runs, reported real and user times are the sum of individual processes and memory is the maximum memory use by any individual process. For piped TKSM runs, the reported time and memory values are captured by GNU Time for the whole pipeline treated as a single process.

Tool	Pipe path	Process	Real time (min)	User time (min)	Memory (GB)	CPUs
TKSM (preprocess)		`scTagger (extract BC)`	1.0	0.9	3.0	1
		`scTagger (LR seg)`	0.8	2.5	1.5	32
		`scTagger (match)`	6.7	159.7	0.9	32
		`minimap2 (badread)`	1.0	0.9	3.2	32
		`badread_error_model`	1.3	1.1	12.5	1
		`badread_qual_model`	3.4	3.3	3.1	1
		`minimap2`	2.4	55.8	6.2	32
		`model_truncation`	0.2	2.3	0.2	32
		`abundance (SC)`	1.1	1.0	2.8	1
		**Total**	**17.8**	**227.3**	**12.5**	**32**
TKSM (core)	head	`transcribe`	1.2	1.2	3.5	1
		`truncate`	0.3	0.3	0.0	1
	path_1	`filter`	0.2	0.2	0.0	1
		`polyA`	0.1	0.1	0.0	1
		`tag`	0.2	0.2	0.0	1
		`single-cell barcode`	0.2	0.2	0.0	1
		`tag`	0.2	0.2	0.0	1
	path_2	`filter`	0.2	0.2	0.0	1
	tail	`PCR`	1.3	1.2	1.4	1
		`flip`	1.4	1.3	0.0	1
		`tag`	1.8	1.7	0.0	1
		`sequence`	9.2	276.5	3.3	32
		**Total**	**16.2**	**283.2**	**3.5**	**32**
TKSM (core; piped)	head	`transcribe`	1.7	1.4	3.5	1
		`truncate`	1.7	0.3	0.0	1
	path_1	`filter`	1.7	0.2	0.0	1
		`polyA`	1.7	0.2	0.0	1
		`tag`	1.7	0.2	0.0	1
		`single-cell barcode`	1.7	0.2	0.0	1
		`tag`	1.7	0.2	0.0	1
	path_2	`filter`	1.7	0.2	0.0	1
	tail	`PCR`	12.2	2.2	1.3	1
		`flip`	12.2	2.2	0.0	1
		`tag`	12.2	2.8	0.0	1
		`sequence`	12.2	325.2	3.3	32
		**Total**	**12.2**	**340.4**	**3.5**	**32**

References

S. L. Amarasinghe, S. Su, X. Dong, L. Zappia, M. E. Ritchie, and Q. Gouil. Opportunities and challenges in long-read sequencing data analysis. *Genome Biology*, 21(1):30, 2020. doi: 10.1186/s13059-020-1935-5.

G. Ebrahimi, B. Orabi, M. Robinson, C. Chauve, R. Flannigan, and F. Hach. Fast and accurate matching of cellular barcodes across short-reads and long-reads of single-cell RNA-seq experiments. *iScience*, 25 (7):104530, 2022. doi: 10.1016/j.isci.2022.104530.

S. Hafezqorani, C. Yang, T. Lo, K. M. Nip, R. L. Warren, and I. Birol. Trans-NanoSim characterizes and simulates nanopore RNA-sequencing data. *GigaScience*, 9(6):giaa061, 2020. doi: 10.1093/gigascience/giaa061.

Y. Hu, L. Fang, X. Chen, J. F. Zhong, M. Li, and K. Wang. LIQA: long-read isoform quantification and analysis. *Genome Biology*, 22(1):182, 2021. doi: 10.1186/s13059-021-02399-8.

F. Karaoglanoglu, C. Chauve, and F. Hach. Genion, an accurate tool to detect gene fusion from long transcriptomics reads. *BMC Genomics*, 23(1):129, 2022. doi: 10.1186/s12864-022-08339-5.

H. Li. Minimap2: pairwise alignment for nucleotide sequences. *Bioinformatics*, 34(18):3094–3100, 2018. doi: 10.1093/bioinformatics/bty191.

Q. Liu, Y. Hu, A. Stucky, L. Fang, J. F. Zhong, and K. Wang. LongGF: computational algorithm and software tool for fast and accurate detection of gene fusions by long-read transcriptome sequencing. *BMC genomics*, 21:1–12, 2020. doi: 10.1186/s12864-020-07207-4.

B. Orabi, E. Erhan, B. McConeghy, S. V. Volik, S. Le Bihan, R. Bell, C. C. Collins, C. Chauve, and F. Hach. Alignment-free clustering of UMI tagged DNA molecules. *Bioinformatics*, 35(11):1829–1836, 2019. doi: 10.1093/bioinformatics/bty888.

B. Orabi, N. Xie, B. McConeghy, X. Dong, C. Chauve, and F. Hach. Freddie: annotation-independent detection and discovery of transcriptomic alternative splicing isoforms using long-read sequencing. *Nucleic Acids Research*, 51(2):e11–e11, 2023. doi: 10.1093/nar/gkac1112.

F. Pedregosa, G. Varoquaux, A. Gramfort, V. Michel, B. Thirion, O. Grisel, M. Blondel, P. Prettenhofer, R. Weiss, V. Dubourg, J. Vanderplas, A. Passos, D. Cournapeau, M. Brucher, M. Perrot, and E. Duchesnay. Scikit-learn: Machine learning in Python. *Journal of Machine Learning Research*, 12: 2825–2830, 2011. URL `http://jmlr.org/papers/v12/pedregosa11a.html`.

H. Sarkar, A. Srivastava, and R. Patro. Minnow: a principled framework for rapid simulation of dscRNA-seq data at the read level. *Bioinformatics*, 35(14):i136–i144, 2019. doi: 10.1093/bioinformatics/btz351.

C. Soneson, Y. Yao, A. Bratus-Neuenschwander, A. Patrignani, M. D. Robinson, and S. Hussain. A comprehensive examination of Nanopore native RNA sequencing for characterization of complex transcriptomes. *Nature Communications*, 10(1):3359, 2019. doi: 10.1038/s41467-019-11272-z.

M. Šošić and M. Šikić. Edlib: a C/C++ library for fast, exact sequence alignment using edit distance. *Bioinformatics*, 33(9):1394–1395, 2017. doi: 10.1093/bioinformatics/btw753.

L. Tian, J. S. Jabbari, R. Thijssen, Q. Gouil, S. L. Amarasinghe, O. Voogd, H. Kariyawasam, M. R. Du, J. Schuster, C. Wang, et al. Comprehensive characterization of single-cell full-length isoforms in human and mouse with long-read sequencing. *Genome Biology*, 22(1):1–24, 2021. doi: 10.1186/s13059-021-02525-6.

R. R. Wick. Badread: simulation of error-prone long reads. *Journal of Open Source Software*, 4(36): 1316, 2019. doi: 10.21105/joss.01316.
